# Editorial: Exercise, physical therapy, and wellbeing in breast cancer patients

**DOI:** 10.3389/fonc.2023.1118718

**Published:** 2023-02-14

**Authors:** Julio C. de la Torre-Montero, Soraya Casla-Barrio, Blanca Herrero-López, José Ángel García-Saénz

**Affiliations:** ^1^ Comillas Pontifical University, San Juan de Dios School of Nursing and Physical Therapy, Madrid, Spain; ^2^ Hospital Gregorio Marañón, Medical Oncology Department, Madrid, Spain; ^3^ Hospital Clínico San Carlos, Medical Oncology Department, Madrid, Spain

**Keywords:** breast cancer, cares, well - being, physical exercice, physical therapy

Cancer diagnoses pose a risk of disease and a challenge for the health system: on the one hand, it is estimated that by 2040 the number of new diagnoses will increase by more than 53%, and on the other hand, in addition to the need for treatments, we have to respond in the form of treatments, which are not only limited to medical treatments or based on pharmacotherapy or radiotherapy, but also include interventions based on nutrition, physical exercise and other motivational interventions from the perspective of psychology and the modification of environmental factors, in addition to the comprehensive care of patients that includes the detoxification of noxious habits, and attention to sexuality (1). In most of this new paradigm of patient care, the family group, and interpersonal relationships play an important role in terms of the changes that must occur in the long term.

Adherence to treatment in these cases is not based on the patient’s motivation to continue pharmacological treatment. Still, it is sometimes based on a change in routines, life habits, and the acquisition of new ones, which include attitude changes, and sometimes result in a decreased need for oral treatments. Body composition in long-term breast cancer survivors taking aromatase inhibitors is improved by aerobic exercise and resistance exercise, in addition to alleviating negative side effects, and patient reports outcomes are also improved (2).

This Research Topic asks, how does the type of well-being recovery program influence the intervention approach is taken, and which type of activity is most effective and adequate for each patient? This special issue brings together three study protocols, two systematic reviews, and one literature review, as well as nine original studies, four of which have a clinical trial design, five are observational studies, and one of which presents questionnaire validation of the quality of life.

Breast cancer, as well as the rest of the oncological diagnoses, presents a challenge, due to its great survival and the possibility of maintaining daily life activities with a high level of well-being and quality of life. Actions aimed at maintaining this quality of life, as well as solving present or potential problems are covered from a multidisciplinary perspective: oncologists, specialist nurses, social workers, physiotherapists, and experts in physical exercise and cancer, in addition to many others. Including patients in primary and secondary prevention and treatment policies as front-line actors ensure they are active in their total recovery. There is already evidence regarding the prevention of breast cancer: some studies show that large cohorts of the population, regarding usual recommendations on physical exercise, lower the risk by 6-10% on those individuals that perform physical activity ranging from 7.5 to 15 MET [the metabolic equivalent of task] per week (3, 4).

One of the most worrying effects for clinicians and patients is usually lymphedema, especially treated from physiotherapy, where, with adequate training, it is expected to reduce the volume of the affected arm, as well as improve quality of life, the strength of grip and resistance in physical exercise performance. The protocol includes moderate to intense exercise sessions, following a specific upper and lower body work plan. (Ramírez-Parada et al.).

Common and known effects derived from post-chemotherapy oral treatments, such as those based on tamoxifen, show that more than 70% of patients show derived symptoms, especially if they are under 40 years of age. Anticipating this type of situation makes it much easier for symptoms to be controlled early and for measures to be taken to mitigate and minimize them (Sung et al.). On the other hand, the importance of behavioral therapies in terms of intrinsic motivation is highlighted to minimize the effects of one of the usual symptoms, such as fatigue, reaching a reduction of this symptom in 77.77% of patients from the start of the intervention until six weeks later. Also, of great importance, the reduction of depressive symptoms was reduced in 55.55% of the study participants (Getu et al.).

Breast tumors have different heterogeneities and one of the less common diagnoses is a neuroendocrine tumor of mammary location (Sun et al). The identification of biological markers that allow responding to less frequent diagnoses is presented as a challenge for researchers, especially when the inhibition of immune control points is not presented as the ideal treatment in these situations, in addition to the fact that chemotherapy is not always effective.

The bone health of patients undergoing hormonal treatment always represents a challenge in the long-term follow-up of patients who are under the effects of the different existing aromatase inhibitor treatments for their diagnosis. Zoledronic acid and Denosumab are the best options in bone resorption and what happens to these patients over time remains to be determined, as well as what aspects could be improved with specific plans for physical exercise, diet, and vitamin D supplementation (Sire et al.). Along the same lines, another systematic review that analyzes the effects of physical exercise and acupuncture shows that the effects of these treatments can minimize pain, in the case of acupuncture, and physical exercise significantly improves activities. of daily life. Secondly, other symptoms such as anxiety, lack of sleep, or fatigue do not present statistically significant improvements (Zhu et al.). Baduanjin is a form of qigong, with eight movements. The purpose of this series of movements is to increase internal energy through exercise and spiritual practice to improve health and fitness. The movements must be executed in a moderate, relaxed, fluid, and consistent manner. The force is only necessary for an instant, when changing movements, maintaining relaxation for the rest of the time. This type of training in 12 weeks could reduce Aromatase Inhibitor side effects: global quality of life and physical functioning scores increased significantly by 12.39 (P < 0.001) and 8.48 (P < 0.001) in the Baduanjin exercise group compared with those in the control group (Liao et al.).

Continuing with the understanding of the different clinical situations that may arise, the study of reproductive hormones, through the measurement of serum levels of reproductive hormones: luteinizing hormone (LH), E2, P, testosterone (T), follicle-stimulating hormone (FSH), and prolactin (PRL) in postmenopausal patients with breast cancer. The expression levels of ER, PR, HER2, and p53 were also determined. The relationships between these receptors and hormones were evaluated in 352 Breast Cancer patients. The results point out that postmenopausal-mediated decreases in serum LH and FSH levels were associated with increased ER and PR expression and decreased HER2 expression (Jiang et al.).

One study analyzed aspects of quality of life and long-term satisfaction in a cohort of 141 patients after reconstruction after breast cancer surgery. The results show that, compared to mastectomy without reconstruction, the latter offers better results for the well-being of patients after the diagnosis of breast cancer. Evaluated at one year and after five years, the best quality of life was evidenced, as well as a better state of well-being in those with psychiatric medication records in their medical history. (Shiraishi et al.).

One of the studies published in this special series analyzes the D-dimer values in the postoperative period of breast cancer surgery and thirteen clinicopathological factors, which were identified and included in the analysis. The distribution of several of these factors between the two groups (pre and postoperative D-dimer levels) was compared. Factors with significantly different distributions between the two groups were identified as potential risk factors for D-dimer variation. The high risk is revealed in patients with a history of diabetes, with complications such as thrombosis. Priority must be paid to preoperative tests to anticipate possible health complications (Wang et al.).

Two studies thoroughly analyze aspects related to physical exercise, of Physical Activity, Fitness, Body Composition, Immunological Biomarkers, and Psychological Parameters During the First Year After Diagnosis in Women with Non-Metastatic Breast Cancer, and the Association of Insomnia, Depressive Disorders, and Mood Disorders as Risk Factors in the general population. The first is a Study Protocol that will quantify daily physical activity and cardiorespiratory fitness in objective measurements in the context of cancer therapy for 12 months after diagnosis. Relationships between exercise, immune status, physical and psychoemotional outcomes, and the clinical course will be studied (Zemlin et al.). The second analyzes a retrospective cohort of 232,108 women diagnosed with insomnia, depressive disorders, and mood disorders. Women with insomnia and hyperlipidemia was associated with an increased risk rate for breast cancer. Insomnia together with the sleeping medication did not produce any more risks than each one alone. Contrary to what is sometimes thought by the common opinion, mood disorders did not appear to be associated with breast cancer diagnosis (Liu et al.).

One of the topics always presented as an area for improvement in research methodology is the statistical analysis methods used to assess the effectiveness of a quality-of-life test. Whether the results of this type of questionnaire are valid in clinical practice, the results of the validation by the Anchor method are presented as an additional solution, offered in the research (Li et al.). The relationship between quality of life and physical activity is already unquestionable. The early physical activity proposed in the APACAN-2 protocol may offer results that improve those interventions that work with patients after the completion of adjuvant therapy (Ginzac et al.). New methods used in diagnosis and therapy are promising and have prospects for future development: surface thermography can be a very good option to monitor rehabilitation programs after mastectomy (Aquino et al.) without adverse effects, offering useful and interesting information for professionals.

Personalized physiotherapy programs, nutritional plans, cognitive interventions, and physical exercise are fundamental not only in nursing care, medical, pharmacological, or radiotherapy treatment. Still, they must go further and integrate each of the aspects that influence the quality of life and well-being of people, patients and their families.

## Author contributions

All authors listed have made a substantial, direct, and intellectual contribution to the work and approved it for publication.
